# A screen for hydroxymethylcytosine and formylcytosine binding proteins suggests functions in transcription and chromatin regulation

**DOI:** 10.1186/gb-2013-14-10-r119

**Published:** 2013-10-24

**Authors:** Mario Iurlaro, Gabriella Ficz, David Oxley, Eun-Ang Raiber, Martin Bachman, Michael J Booth, Simon Andrews, Shankar Balasubramanian, Wolf Reik

**Affiliations:** 1 Epigenetics Programme, Babraham Institute, Babraham Research Campus, Cambridge CB22 3AT, UK; 2 Centre for Haemato-Oncology, Barts Cancer Institute, Charterhouse Square, London EC1M 6BQ, UK; 3 Proteomics Research Group, The Babraham Institute, Babraham Research Campus, Cambridge CB22 3AT, UK; 4 Department of Chemistry, University of Cambridge, Lensfield Road, Cambridge CB2 1EW, UK; 5 Cancer Research UK, Cambridge Research Institute, Li Ka Shing Centre, Robinson way, Cambridge CB2 0RE, UK; 6 School of Clinical Medicine, The University of Cambridge, Addenbrooke’s Hospital, Hills Road, Cambridge CB2 0SP, UK; 7 Bioinformatics Group, Babraham Institute, Babraham Research Campus, Cambridge CB22 3AT, UK; 8 Centre for Trophoblast Research, University of Cambridge, Cambridge CB2 3EG, UK; 9 1Wellcome Trust Sanger Institute, Cambridge CB10 1SA, UK

## Abstract

**Background:**

DNA methylation (5mC) plays important roles in epigenetic regulation of genome function. Recently, TET hydroxylases have been found to oxidise 5mC to hydroxymethylcytosine (5hmC), formylcytosine (5fC) and carboxylcytosine (5caC) in DNA. These derivatives have a role in demethylation of DNA but in addition may have epigenetic signaling functions in their own right. A recent study identified proteins which showed preferential binding to 5-methylcytosine (5mC) and its oxidised forms, where readers for 5mC and 5hmC showed little overlap, and proteins bound to further oxidation forms were enriched for repair proteins and transcription regulators. We extend this study by using promoter sequences as baits and compare protein binding patterns to unmodified or modified cytosine using DNA from mouse embryonic stem cell extracts.

**Results:**

We compared protein enrichments from two DNA probes with different CpG composition and show that, whereas some of the enriched proteins show specificity to cytosine modifications, others are selective for both modification and target sequences. Only a few proteins were identified with a preference for 5hmC (such as RPL26, PRP8 and the DNA mismatch repair protein MHS6), but proteins with a strong preference for 5fC were more numerous, including transcriptional regulators (FOXK1, FOXK2, FOXP1, FOXP4 and FOXI3), DNA repair factors (TDG and MPG) and chromatin regulators (EHMT1, L3MBTL2 and all components of the NuRD complex).

**Conclusions:**

0ur screen has identified novel proteins that bind to 5fC in genomic sequences with different CpG composition and suggests they regulate transcription and chromatin, hence opening up functional investigations of 5fC readers.

## Background

Levels of 5hmC in DNA (and where known 5fC and 5caC) vary between different mammalian tissues and are highest in ES cells and neural tissues [1-5]. In situations where oxidative derivatives of 5mC are implicated in demethylation of DNA, such as in pluripotent stem cells, early embryos and germ cells, there may be rapid turnover of these modifications through a combination of further oxidation, DNA replication, excision repair by TDG, and potentially deamination or decarboxylation [6-8]. In other tissues, especially those with non-dividing cells such as neural tissues, the modifications could potentially be more stable and might thus be used as epigenetic signals for genome function [9-11]. A variety of proteins that bind to histone modifications or to methylated DNA (methyl binding domain proteins (MBDs)) have been described and have a role in interpreting these epigenetic signals for the regulation of transcription, replication, DNA repair or other functions of the genome [12-14]. Recently, MBD3 and MECP2 have been shown to be able to bind 5hmC (MBD3 weakly so) in addition to 5mC, opening up the possibility that these proteins may also be able to interpret the 5hmC signal, for example, in the regulation of transcription or chromatin [15,16]. A recently published unbiased screen [11] has identified and validated a number of proteins with specific binding to 5mC and its oxidised forms but the use of a single DNA probe overlooks the possibility that proteins in a cellular context might have a combined preference for both DNA modification and sequence context. Indeed some of the proteins identified as specific for a DNA modification are cell-type specific, suggesting a complex protein interaction network operating in modulating the intrinsic ability to bind to DNA modifications.

## Results and discussion

**Figure 1:**
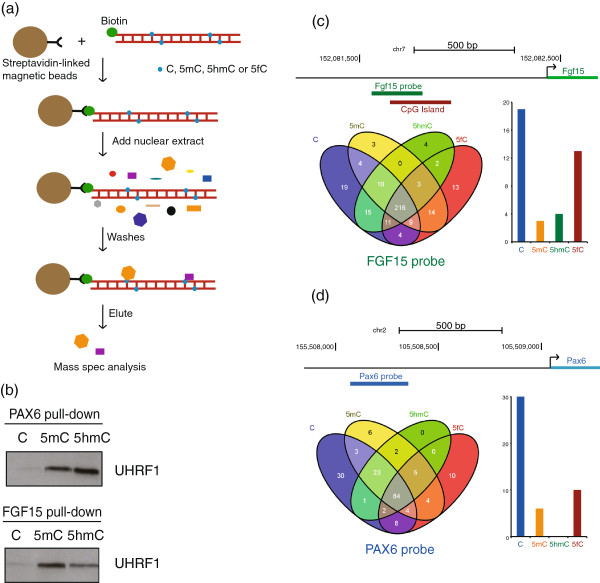
**A mass spectrometry-based method for detection of 5-formylcytosine binding proteins. (a)** Schematic representation of the pull-down method. DNA oligonucleotides corresponding to the promoter regions of the *Pax6* (280 bp) and *Fgf15* (248 bp) genes were obtained by PCR with biotinylated primers and using dATP, dGTP. dTTP and either dCTP, dmCTP, dhmCTP or dfCTP. DNA was then incubated with Streptavidin-linked beads and with nuclear extract from mouse ES cells. Bound fraction was then eluted and analysed by mass spectrometry. **(b)** Western blot showing presence of UHRF1 in the protein fraction captured by methylated and hydroxymethylated probes (both *Fgf15* and *Pax6*) compared to umodified DNA. **(c, d)** Venn diagrams and histograms showing distribution of significantly enriched proteins binding to differentially modified *Fgf15* probe (CpG: 14; non-CpG: 69, %CpGs: 11.3%) and *Pax6* probe (CpG: 8; non-CpG: 44; %CpG: 5.7%) with schematic representation of their genomic position.

We established a proteomics screen for C, 5mC, 5hmC or 5fC binding proteins based on modifications of published protocols [17]. Briefly, PCR probes were made corresponding to the promoter regions of the *Pax6* and *Fgf15* genes (relative position to the gene is shown in Figure 1c and 1d). Both of these genomic regions are enriched for 5hmC in mESCs, and their corresponding gene expression is associated with changes in the relative levels of 5mC/5hmC in control relative to Tet1 siRNA-treated cells [18]. Modified cytosines were incorporated during PCR and the probes were then incubated with nuclear protein extracts from mESCs (E14 ES cells cultured in Serum/LIF conditions). Proteins which bound to the probes were eluted and identified by mass spectrometry (Figure 1a and full table in Additional file 1). We initially verified whether the screen was able to enrich the previously known 5mC/5hmC binder NP95/UHRF1 [19]. Indeed the western blot in Figure 1b shows specific binding of the protein to both modifications. Our mass spectrometry results also confirmed the recently identified proteins specifically binding to C (KDM2B, CXXC5, BCOR) and 5mC (RFX1, MBD4) (Additional file 1 and [11]).

**Figure 2:**
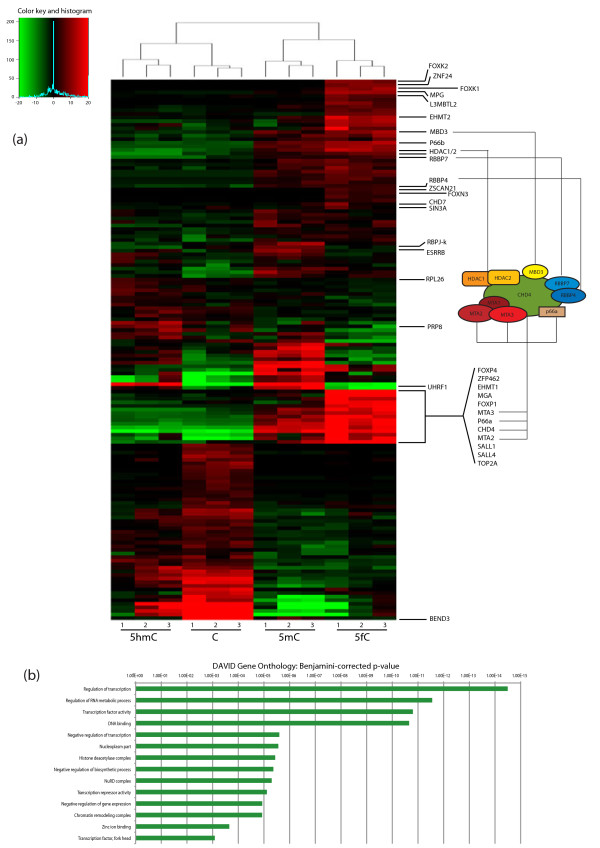
**5-formylcytosine specific binders to *Fgf15 *probe are enriched for transcription factors and chromatin regulators. (a)** Heatmap representation of the relative protein enrichment on the *Fgf15* probe. Spectral count values for each replicate were analysed by testing the sample groups using a non-parametric Kruskal-Wallis *t*-test with a *P* value cutoff of 0.1. For heatmap display, additional filters for the size of absolute change between group means were applied, and the data for each gene were normalised by subtracting the median value for that gene across all experiments from the individual values. A cartoon highlights presence of all the component of the main core of the NuRD complex among the 5fC binders. **(b)** Functional annotation enrichment analysis performed on 5fC binders using DAVID shows enrichment for transcription (mainly zinc-binding factors) and chromatin regulators. Results are expressed with their corresponding Benjamini-corrected *P* value.

**Figure 3:**
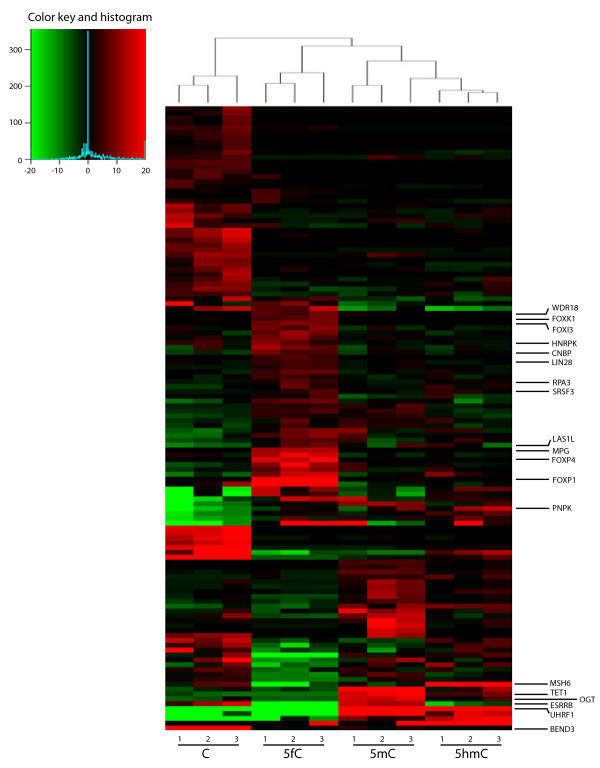
**Relative protein enrichment in pull-downs with the *Pax6 *probe.** Heatmap representation of the relative protein enrichment on the *Pax6* probe. Spectral count values for each replicate were analysed by testing the sample groups using a non-parametric Kruskal-Wallis *t*-test with a *P* value cutoff of 0.1. For Heatmap display, additional filters for the size of absolute change between group means were applied, and the data for each gene were normalised by subtracting the median value for that gene across all experiments from the individual values.

Having established a screen that was robust and identified known binders of both 5mC and 5hmC, we systematically evaluated all binding proteins and included 5fC modified targets in the screen (Additional file 1, Figure 1c and d, Figures 2 and 3). Pull-downs were performed in triplicate for each DNA modification with both *Pax6* and *Fgf15* probes, and resulting values were analysed using a non-parametric Kruskal-Wallis ANOVA with a threshold sufficient to identify proteins where the replicates for one modification were consistently the most enriched against a random set of enrichments in the other pull-downs. The Venn diagrams in Figure 1c and 1d include only proteins with significant enrichment and show binding distribution to differentially modified probes. A detailed representation of relative binding of proteins to each modification in each target sequence is shown in Figures 2a and 3. Heatmaps were generated by unsupervised hierarchical clustering of the mass spectral counts for each protein (horizontal lines) binding to each modification in three replicate pull-downs, normalised by row mean subtraction. Protein enrichment is indicated in red (highly enriched) to green (under-enriched relative to mean). Some of the candidate proteins are highlighted on the right side of the heatmaps and the full list is shown in Additional file 2.

**Figure 4:**
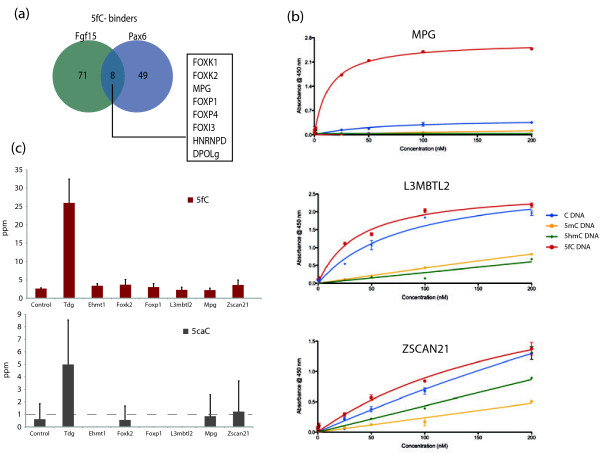
**Validation and functional analysis of 5fC binding proteins. (a)** Venn diagram illustrating overlap between 5fC specific binders identified by the two different probes used. **(b)** ELISA assays performed with purified recombinant MPG, L3MBTL2 and ZSCAN21 proteins and differentially modified *Fgf15* probe (blue = unmodified DNA; yellow = methylated DNA; green = hydroxymethylated DNA; red = formylated DNA). MPG (specifically bound to 5fC on both probes) shows strong selective binding for formylated DNA (Kd = 13.4 ±1.4 nM). L3MBTL2 (Kd = 37.1 ±5.6 nM for 5fC and Kd = 81.2 ±18.8 nM for C) and ZSCAN21 show preference of binding. This could reflect the difference in DNA interaction between an enzyme and transcriptional regulators. **(c)** Mass spectrometry analysis of global 5-formylcytosine (red bars) and 5-carboxycytosine (grey bars) levels in J1 ES cells after three rounds of knockdown of potential 5fC binders, compared to cells transfected with non-targeting siRNA. Bars show average of four biological replicates with corresponding standard deviation, expressed as the number of modified cytosines per million of all cytosines. Dotted line indicates the limit of accurate quantification.

Of interest were proteins that bound only to unmodified C, such as BEND3, USF1, USF2, CXXC5 and KDM2B, perhaps reflecting a binding architecture that is disrupted by modifications on the DNA. Among proteins that showed specificity for 5mC are previously identified methyl-CpG binding proteins like MBD4 and RBPJ [20,21], but also TET1, OGT and interestingly a key pluripotency regulator ESRRB [22], which has not been previously identified as a 5mC binding protein (Figures 2 and 3). Only few proteins showed a strong preference for 5hmC (such as RBM14, PRP8 and RPL26 on *Fgf15*, MSH6 and PNKP on *Pax6* probe, respectively). Similarly to Spruijt et al. [11] we also did not find MBD3 binding to 5hmC with higher affinity than to 5mC (as was previously reported by Yildirim et al. [15]). Instead, MBD3 showed selective binding to 5mC in the *Pax6* target and to 5mC/5fC in the *Fgf15* target, in agreement with Spruijt et al. where MBD3 at high concentrations had higher affinity to 5mC [11,23]. Our screen revealed that more proteins bind uniquely to 5fC than to other DNA modifications (barplots in Figure 1c and 1d). Notably, 21 proteins were found exclusively bound to the 5fC probes - 11 on the *Fgf15* probe (among which are TDG, SIX4, ZSCAN21 and ZKSCAN3), 8 on the *Pax6* probe (including MPG, FOXP4 and CRSP2) and 2 to both probes (FOXK2 and FOXI3). Many more proteins bound to 5fC preferentially (Additional files 1 and 2 and Figure 4a).

Gene ontology term enrichment comparing modification specific binders to the full set of identified proteins showed highly significant groups enriching with relevance to gene transcription and chromatin regulation among 5fC binders on the *Fgf15* probe (Figure 2b). Association of 5fC with repressive transcription complexes was a surprising finding where, notably, all members of the core NuRD complex were enriched in the group of 5fC specific binding proteins (Figure 2a), although it is likely that some of the members of the complex are not direct 5fC binders but are enriched by secondary protein-protein interactions. This indicates that 5fC is more likely to be associated with gene repression. Interestingly, many of the proteins enriched for 5fC at the *Fgf15* probe were enriched for 5mC too, as seen by the hierarchical clustering, strengthening the potentially repressive properties of 5fC especially in the context of a CpG island sequence. This was not the case with the *Pax6* probe, which is not a CpG island (Figure 3). It remains to be seen if the presence of 5fC in CGIs has inhibitory functions, especially in the process of cell differentiation. Clustering of proteins enriching on the *Pax6* probe did not result in a similar grouping of 5fC and 5mC enriching repressive proteins and the GO analysis showed no significant enrichment for repressive complexes indicating that the DNA sequence of *Pax6* might lack the DNA signatures of a typical CpG island therefore may not result in an inhibitory transcriptional signal in the presence of 5fC. While our experimental system made use of a promoter CpG island (in *Fgf15*) these insights may also be applicable to intragenic CpG islands, which can have higher levels of DNA modifications [24]. The association between 5-formylcytosine and transcription has been investigated recently, resulting in its linkage variously with active or poised genes [25-27]. Our results potentially reinforce the idea that depending on context 5fC could have positive or negative effects on transcription. Nevertheless, some of the 5fC specific proteins were enriched with both DNA probes and are shown in Figure 4a. This comparison strongly suggests that Fork head box domain containing proteins have 5fC binding properties. Gene ontology results for the other cytosine modifications for the two probes are included as Additional files 3 and 4.

In order to validate some of these candidate proteins for 5fC binding specificity, we performed ELISA with purified recombinant proteins and differentially modified *Fgf15* probes. His-tagged isoforms of MPG, L3MBTL2 and ZSCAN21 were expressed in Sf9 insect cells using a Baculovirus system, and purified by immobilised metal ion affinity chromatography (IMAC). We found that all three proteins bound with higher affinity to 5fC compared to the other modifications on the DNA (Figure 4b). MPG is one of the proteins common for both DNA targets and showed a strong binding preference for 5fC. In a recent study MPG was identified as a 5hmC specific binder but the data actually show some binding to 5fC as well [11], and considering different culturing conditions of ES cells (2i/LIF), post-translational modifications might modulate the binding of some proteins to their target [28]. Finally, we considered the possibility that the 5fC binding proteins might have a role in the excision of 5fC similar to TDG. We therefore tested this hypothesis by RNAi in ES cells (Figure 4c, Additional files 5 and 6). While knockdown of TDG (which is known to excise 5fC and 5caC [29,30]) resulted in increase of 5fC and 5caC (as measured by mass spectrometry), knockdown of the other candidates had no effect. We therefore conclude that the majority of 5fC binding proteins identified in this screen are less likely to metabolize 5fC, instead they are more likely to recognize 5fC as an epigenetic signal.

The preferential binding of TET1 to both 5mC (more strongly) and 5hmC, compared to C (Figure 3) was interesting since the CXXC domain of TET1 has been shown to differ from that of other CXXC domain-containing proteins, lacking a typical 'KFGG’ motif found in most of the family, with some studies showing its inability to bind DNA [31], and others suggesting that this peculiarity allows it to bind not only to unmodified and methylated DNA, but also to hydroxymethylated DNA [32,33]. This opens the possibility that the binding could be influenced by sequence context or protein modifications.

It was of particular interest that our screen identified a higher number of proteins that appear to preferentially bind to 5fC (Figure 1c,d) rather than to other modifications, an observation also reported in Spruijit et al. [11]. It is not immediately intuitive why there should be more proteins binding to 5fC than to 5hmC. Of course this could depend on the tissue analysed and there might be more 5hmC binding proteins in neural cell types, for example, where the modification is relatively prevalent. Intriguingly, FOXK2 in addition to being a member of the forkhead box transcription factor family has been shown to bind to T:G mismatches in DNA but no enzymatic activity has been identified [34]. Another member of this family, FOXP1, a key transcriptional regulator in B cells and lung development was also identified as strong and specific 5fC binder in our screen. Recent reports have shown that an ES cell-specific isoform of FOXP1 is implicated in pluripotency regulation in ESCs by stimulating expression of pluripotency-related genes like *Oct4*, *Nanog* and *Nr5a2*[35]. FOXP4, also enriched on both 5fC probes, is involved in development of the lung and is known to form homodimers and heterodimers with FOXP1, and to interact with NuRD components [36]. FOXK1 is a transcriptional regulator involved in myogenic regulation [37], while relatively little is known about the function of mouse FOXI3. Another transcription factor that appears to bind specifically to 5fC in our screen is ZSCAN21, a strong transcriptional activator that plays a role in both male and female meiosis [38,39]. The final protein in this category of transcriptional regulation linked with DNA repair is MPG, which is a base excision repair glycosylase known to excise modified bases resulting from alkylation damage. MPG was a highly specific binder for 5fC in our screen, while the human isoform bound strongly to 5fC in a HeLa sample extract providing an additional layer of confidence (data not shown); MPG has been identified as a interacting partner of MBD1 [40] and, intriguingly, its methyl-purine glycosylase domain structurally resembles the formyl transferase, C-terminal-like domain (IPR011034).

The last category of 5fC binders makes interesting connections with chromatin regulation through the polycomb and histone methylation pathways. In addition to the previously mentioned correlation between 5fC and the NuRD complex, components of another chromatin regulator complex, E2F6.com-1, were also identified as 5fC binders. In addition to MGA and CBX3, we isolated and verified L3MBTL2 as a 5fC binder, which is a putative polycomb protein which may bind to modified histones, while EHMT1 is a euchromatin histone methyltransferase that methylates H3K9 to H3K9me1 and me2, potentially providing a link between modifications in euchromatin that are intermediates between transcriptional repression and activation [41,42].

## Conclusions

We have established a relatively simple and robust screen for proteins that bind 5hmC and 5fC in DNA. 5fC has so far been found in early embryos, embryonic stem cells and brain cortex, as well as in other major mouse organs like spleen, pancreas and liver [43]. The distribution of 5fC in ESCs depends on TDG and recent studies have linked it with the regulation of transcription, variously associated with active or poised genes [25-27]. Our screen has identified 5fC-binding proteins with functions in transcription and in chromatin regulation, particularly involving forkhead box domain transcriptional regulators and the NuRD complex. This suggests that 5fC may be both an intermediate in demethylation and an epigenetic signal in its own right. The dual potential of some of the proteins we have identified (FOXK2 in transcription and DNA repair, EHMT1 mediating between 5fC and H3K9 methylation) is particularly interesting and warrants future functional investigations.

## Methods

### Cell lines and cell culture

E14 ES cells (derived from the E14 cell line strain 129P2/OlaHsd) were grown on a γ-irradiated pMEF feeder layer at 37°C and 5% CO_2_ in complete ES medium (DMEM 4,500 mg l^-1^ glucose, 4 mM l-glutamine and 110 mg l^-1^ sodium pyruvate, 15% fetal bovine serum, 100 U of penicillin/100 μg of streptomycin in 100 mL medium, 0.1 mM non-essential amino acids, 50 μM β-mercaptoethanol, 10^3^ U LIF ESGRO).

### Nuclear extraction

Cells were washed with 1× PBS solution, detached adding trypsin at 37°C to the culture plate and centrifuged at 300 × g for 4 min. The pellet was then washed in ice-cold 1× PBS twice and resuspended gently in 5 volumes of ice-cold 1 Cytoplasmic Lysis Buffer (Chemicon International^®^) containing 0.5 mM DTT and 1/1,000 dilution of supplied protease inhibitor Cocktail. The solution was incubated on ice for 15 min, centrifuged at 300 × g for 5 min at 4°C, and the pellet was resuspended in two volumes of ice-cold 1× Cytoplasmic Lysis Buffer. Cells were lysed using a 27-gauge needle and the nuclear fraction was isolated from the cytosolic portion by centrifugation at 8,000 × g for 20 min at 4°C. Finally, the pellet was resuspended in two-thirds of the original cell pellet volume of ice-cold Nuclear Extraction Buffer (Chemicon International^®^) containing 0.5 mM DTT and 1/1,000 dilution of supplied protease inhibitor cocktail, incubated on orbital shaker for 60 min at 4°C, and centrifuged at 16,000 × g for 5 min at 4°C. The nuclear extract was then aliquoted and stored at -80°C.

### DNA probes

The probes were obtained by PCR amplification of genomic region corresponding to the promoters of *Pax6* (280 bp) and *Fgf15* (248 bp) genes using DreamTaq™ DNA Polymerase (Fermentas). The primers used in the reaction were:

*Pax6*-F (Biotinylated): ATTCCCAAAGCAAGCAGAAG

*Pax6*-R: ACTGTTGACTTTGTGGCCTAGA

*Fgf15*-F (Biotinylated): TTTCTTTCAGGCAGGGGAAT

*Fgf15*-R: TTGAGAAGGGTGGACTGACC

### Pull-down

The pull-down assay was carried out using Dynabeads^®^ M-280 Streptavidin (Invitrogen™). For each sample, 2 μL of beads were washed in buffer PBT (1× PBS, 0.1% Triton X-100), and incubated with 50 ng of biotinylated DNA in 200 uL of PBS, overnight at 4°C. The beads were then washed three times in PBT and twice in buffer D-T (0.2 mM EDTA, 20% Glycerol, 20 mM Hepes-KOH pH 7.9, 0.1 M KCl, 1 mM DTT, 1 mM protease inhibitor PMSF, 0.1% Triton X-100), and incubated with 50 μg of nuclear extract for 15 min at 4°C in incubation buffer (0.05 mM EDTA, 5% Glycerol, 5 mM Hepes-KOH pH 7.9. 150 mM KCl, 1 mM DTT, 1 mM protease inhibitor PMSF, 0.025% Triton X-100 in PBS). The beads were washed six times in Buffer D-T, once in PBS and eluted in 1X LDS Loading buffer boiling at 95°C for 5 min. The eluted fraction was separated from the beads and finally analysed by mass spectrometry.

### RNAi knockdown of Mpg, Tdg, L3mbtl2, Zscan21, Ehmt1, FoxK2 and FoxP1 in ES cells

Transfections of Dharmacon siGENOME SMARTpool against mouse *Tdg* (catalogue number M-040666-01; gaagugcaguauacauuug, gaguaaagguuaagaacuu, caaagaagauggcuguuaa, gcaaggaucugucuaguaa) and siGENOME ON-TARGETplus siRNA against *Mpg* (catalogue no. J-060513-11; ccggcuaggaccagaguuu), *L3mbtl2* (catalogue no. J-065321-12; uuacugacuggaagagcua), *FoxP1* (catalogue no. J-065400-09; gagcaugcgcuggacgaua), *Ehmt1* (catalogue no. J-059041-12; gagcacagguggauccgaa), *Zscan21/Zipro1* (catalogue no. J-048225-09; cuagagauaucccguaaga), *FoxK2* (catalogue no. J-064514-12; ccagagcucaagcgaguua) were done with Lipofectamine 2000 according to the manufacturer’s instructions. Cells were harvested after three rounds of transfection for DNA/RNA isolation.

### Mass spectrometry

Eluted proteins were run a short distance (approximately 5 mm) into an SDS-PAGE gel, which was then stained with colloidal Coomassie stain (Imperial Blue, Invitrogen). The entire stained gel pieces were excised, then destained, reduced, carbamidomethylated and digested overnight with trypsin (Promega sequencing grade, 10 ng/μL in 25 mM ammonium bicarbonate) as previously described [44]. Aliquots of each of the resulting tryptic digests were analysed by LC-MS/MS on a system comprising a nanoLC (Proxeon) coupled to a LTQ Orbitrap Velos mass spectrometer (Thermo). LC separation was achieved on a reversed-phase column (Reprosil C18AQ, 0.075 × 150 mm, 3 μm particle size), with an acetonitrile gradient (0-35% over 60 min, containing 0.1% formic acid, at a flow rate of 300 nL/min). The mass spectrometer was operated in data-dependent acquisition mode, with an acquisition cycle consisted of a high resolution precursor ion spectrum over the m/z range 350–1,500, followed by up to 20 CID spectra (with a 60 s dynamic exclusion of former target ions). Mass spectrometric data were searched against a database generated from the mammalian entries in Uniprot 2011.09 by concatenation of the forward and reversed sequences, using Mascot (Matrix Science) and the search results were processed using Scaffold software (Proteome Software Inc.). Criteria for protein identification were: minimum of two peptides, each with a probability of >50% and an overall protein probability of >99%, which gave a protein false discovery rate of 0.4%. The mass spectrometry proteomics data have been deposited to the ProteomeXchange Consortium [45] via the PRIDE partner repository [46] with the dataset identifier PXD000524.

### Western blot

Pulled-down proteins were eluted from beads in LDS Loading buffer, boiled and run on NuPAGE^®^ Novex 4-12% Bis-Tris Gel 1.0 mm (Novex^®^). Proteins were transferred on a nitrocellulose membrane using iBlot^®^ Blotting System (Life Technologies), membrane was blocked overnight in PBS-0.1%Tween (PBST) containing 5% BSA (blocking buffer). Primary antibody incubation was done at room temperature for 2 h with a rabbit polyclonal anti-UHRF1 Antibody (Santa Cruz M-132: sc-98817). Membrane was washed in PBST and incubated with HRP conjugated anti-rabbit secondary antibody in blocking buffer. HRP conjugates were detected with enhanced chemiluminescence (ECL, Amersham Biosciences).

### Enzyme-linked immunosorbent assay (ELISA)

All binding reactions were carried out in buffer Z containing 20 mM TRIS HCL (pH 7.5), 150 mM NaCl, 20 mM KCl, 0.02% IGEPAL and 1 mM dithiothreitol. A Highbind Streptaplate (Roche) was blocked with 1 × PBS containing 3% BSA prior reaction. Subsequently, 50 μL of a 50 nM solution of biotinylated DNA were added per well and allowed to attach for 30 min at 37°C with gentle shaking. Wells were then washed three times with buffer Z. The proteins were diluted in buffer Z and 50 μL were added to each well. After incubation for 1 h at room temperature, plates were washed three times with buffer Z. For detection, 50 μL of mouse polyclonal anti-His tag antibody (Thermo Scientific) at 1:500 dilution in buffer Z were added per well and incubated for 1 h at room temperature. After washing three times with buffer Z, a polyclonal HRP-conjugated sheep anti-Mouse IgG antibody (GE Healthcare) diluted 1:2,000 in buffer Z was added and incubated for 30 min at room temperature. Wells were washed three times with buffer Z and peroxidase activity detected by adding 50 μL of TACS-Sapphire (Trevigen). Reactions were stopped by the addition of 50 uL of a 1 M HCL solution. Absorbance at 450 nm was measured using a SPECTROstar Nano (BMG Labtech). The equilibrium dissociation constants (Kd) for the protein-DNA interaction were determined by non-linear regression by fitting to a hyperbolic binding curve.

### Purification of recombinant MPG, L3MBTL2 and ZSCAN21 from Baculovirus infected Sf9 cells

Coding sequences for the proteins MPG, L3MBTL2 and ZSCAN21 (Source BioScience) were cloned into Gateway^®^ entry vector pENTR223.1 using SfiI restriction sites. CDS were then cloned into destination vector pDEST10 using Gateway^®^ LR Clonase II mix (Invitrogen) and following manufacturer’s instructions. Resulting vectors were used to transform MAX Efficiency^®^ DH10Bac™ cells (Invitrogen). Positive clones were selected by blue-complementation and correct insertion of sequence of interest was confirmed by PCR. Resulting bacmids were then transfected into Sf9 cells using Cellfectin^®^ II Reagent (Invitrogen). Baculoviruses were then amplified and Sf9 cells expressing the proteins of interest were then harvested at 48, 72 and 96 h post infection for protein expression analysis. Cells pellets were resuspended in Lysis Buffer (50 mM NaH_2_PO_4_, 300 mM NaCl, 10 mM imidazole, 1% Triton and protease inhibitors), incubated on ice for 10 min and centrifuged at 10,000 × g for 10 min at 4°C. Cell lysates were filtered through a 0.2 μm filter and loaded on 1 mL HisTrap HP column (GE Healthcare) equilibrating with buffer A (50 mM NaH_2_PO_4_, 300 mM NaCl, 20 mM imidazole), washed with 10 column volumes of buffer A added with 40 mM imidazole. Proteins were eluted with a gradient of 40–500 mM imidazole over 20 column volumes. Protein samples were dialysed against storage buffer (25 mM Tris–HCl pH 7.5 10% glycerol, 150 mM NaCl, 1 mM DTT).

### Data analysis

Spectral count values from LC-MS/MS were analysed by testing the sample groups using a non-parametric Kruskal Wallis *t*-test with a *P* value cutoff of 0.1, which was determined to be sufficient to identify any group where the most extreme values all fell within that group, regardless of how the values were distributed across the other groups.

### Gene ontology

Functional annotation enrichment analyses were performed using The Database for Annotation, Visualization and Integrated Discovery (DAVID) v6.7 [47-49].

### Mass spectrometry of nucleosides

Quantitation of nucleosides in genomic DNA was done essentially as described previously [27] except that a Q-Exactive mass spectrometer (Thermo) fitted with an UltiMate 3000 RSLCnano HPLC (Dionex) was used and one additional transition 272.1 >156.0404 (caC) was monitored. Results are expressed as % or ppm of total unmodified and modified cytosines.

## Competing interests

The authors declare that they have no competing interests.

## Authors’ contributions

MI and GF conceived the study and analysed the data. MI performed the experiments. DO carried out mass spectrometric analysis of pull-downs. SA performed statistical analysis. ER performed ELISA experiments. MJB helped with generation of the probes. MB analysed 5fC levels by mass spectrometry. WR and SB conceived the study; MI, GF and WR wrote the manuscript. All authors have interpreted the data, read and approved the manuscript.

## Supplementary Material

Additional file 1 **Complete pull-down data.** Excel file with table showing all proteins identified by mass spectrometry in the three replicates, with their corresponding spectral counts. Sheet 1 lists proteins identified by the *Fgf15* probe, sheet 2 lists proteins identified by the *Pax6* probe. Click here for file

Additional file 2 **Pull-down data relative to all proteins with significant enrichment.** Excel file with table showing all proteins that passed the significance test, with their corresponding spectral counts in the three replicates and *P* value. Sheet 1 lists proteins identified by the *Fgf15* probe, sheet 2 lists proteins identified by the *Pax6* probe. Click here for file

Additional file 3 **DAVID Gene ontology analysis on proteins enriched for C, 5mC and 5hmC on the *Fgf15 *probe.** Enrichment for 5fC is shown in Figure 2b. Results are expressed with their corresponding Benjamini-corrected *P* value. Click here for file

Additional file 4 **DAVID Gene ontology analysis on proteins enriched for C, 5mC and 5hmC on the *Pax6 *probe.** 5fC binding proteins showed no significant term enrichment. Results are expressed with their corresponding Benjamini-corrected *P* value. Click here for file

Additional file 5 **Knockdown efficiency. Bar plots showing knockdown efficiency in mESC.** Dark grey bars indicate mRNA levels in the knockdown samples, light grey in the control samples (transfected with non-targeting siRNA). Click here for file

Additional file 6 **Mass spectrometry of nucleosides data.** Excel file showing mass spectrometry data from the knockdown samples (four biological replicates each). Click here for file
