# Supplementary figures and images for: A screen for hydroxymethylcytosine and formylcytosine binding proteins suggests functions in transcription and chromatin regulation
Source: Genome Biol. 2013 Oct 24;14(10):R119. doi: 10.1186/gb-2013-14-10-r119 (PMC4014808; doi:10.1186/gb-2013-14-10-r119)

## Fgf15 - C binders

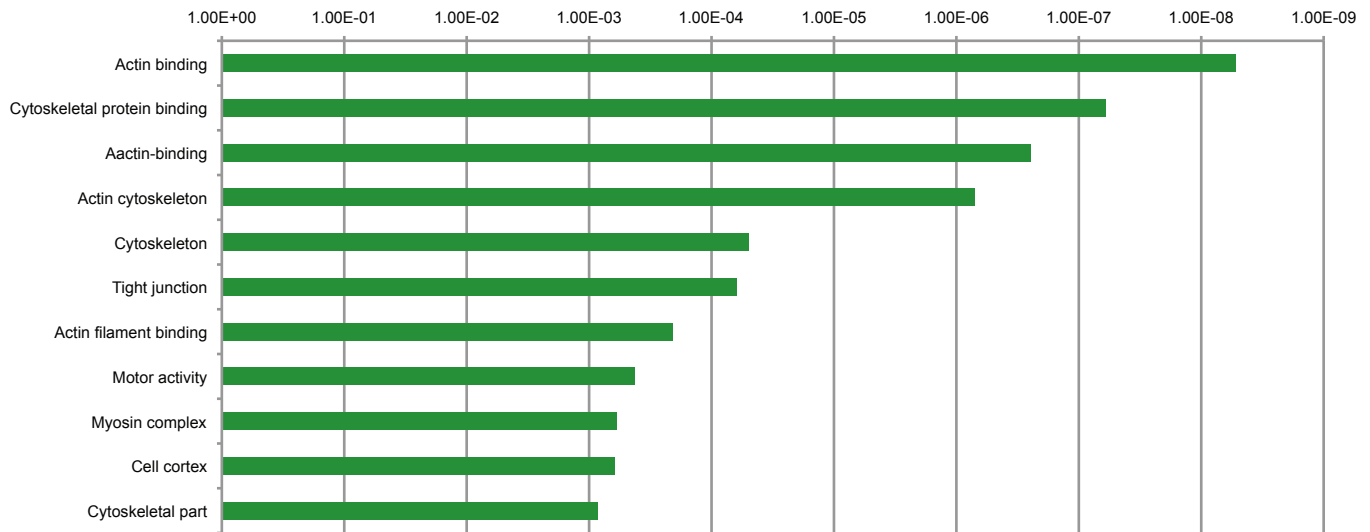

## Fgf15 - 5mC binders

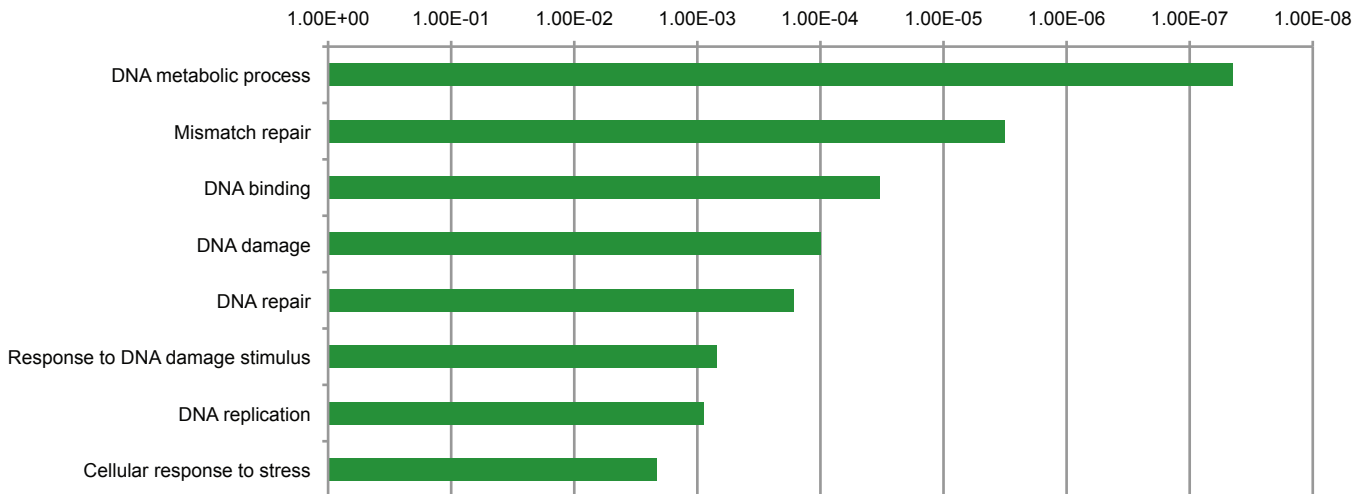

## Fgf15 - 5hmC binders

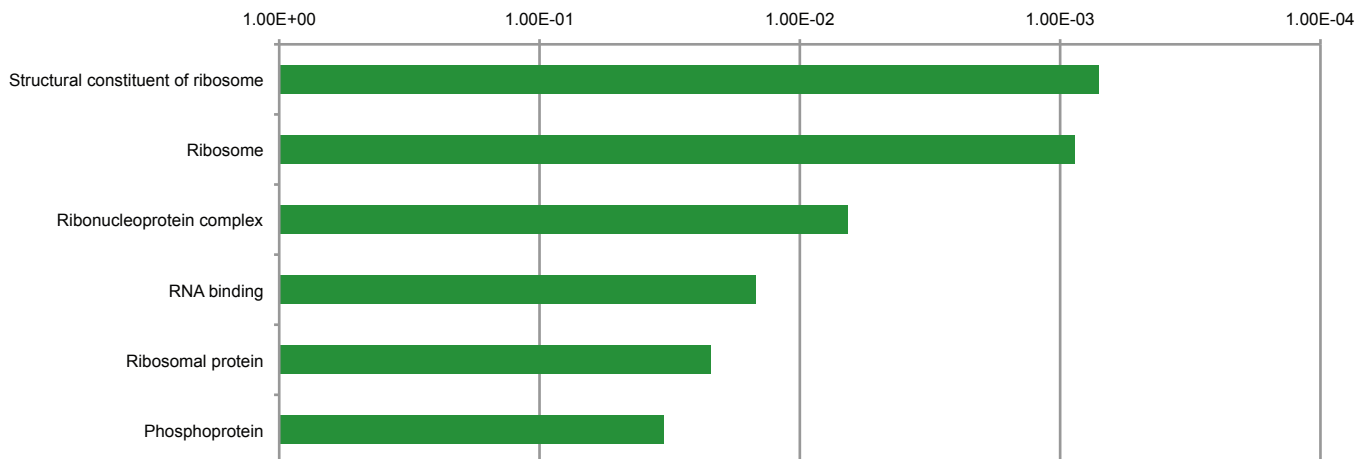

Supplement: Additional file 3 — DAVID Gene ontology analysis on proteins enriched for C, 5mC and 5hmC on the Fgf15 probe. Enrichment for 5fC is shown in Figure 2b. Results are expressed with their corresponding Benjamini-corrected P value. [file gb-2013-14-10-r119-S3.pdf]

## Pax6 - C binders

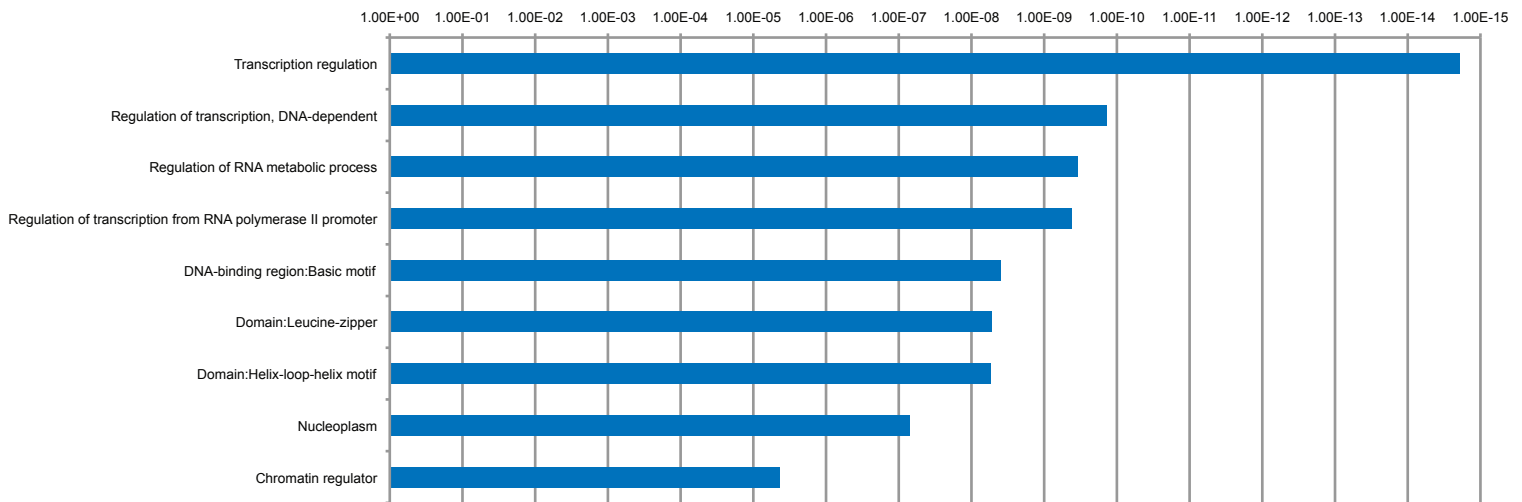

## Pax6 - 5mC binders

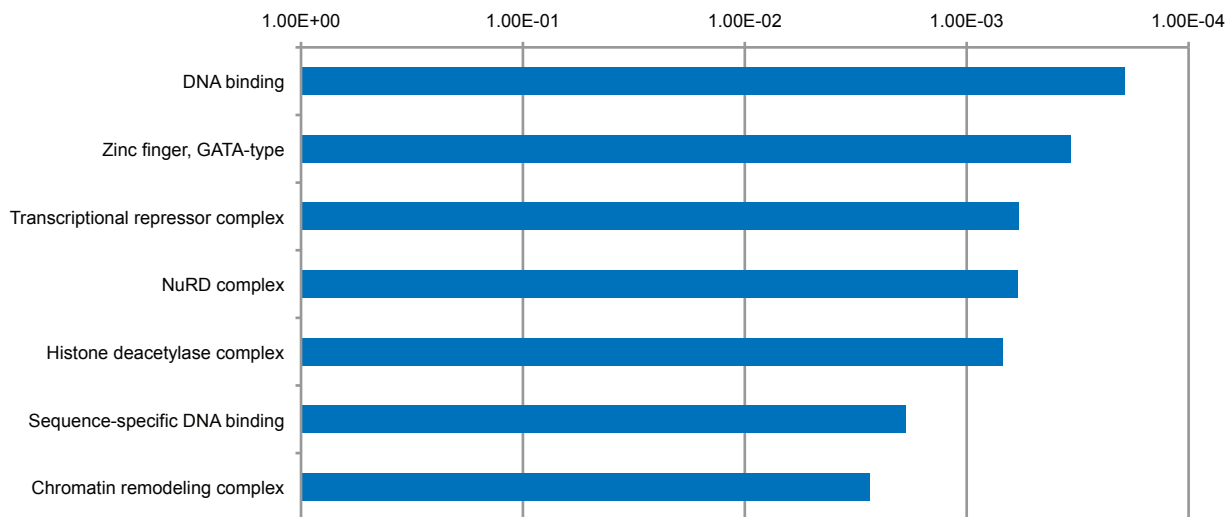

## Pax6 - 5hmC binders

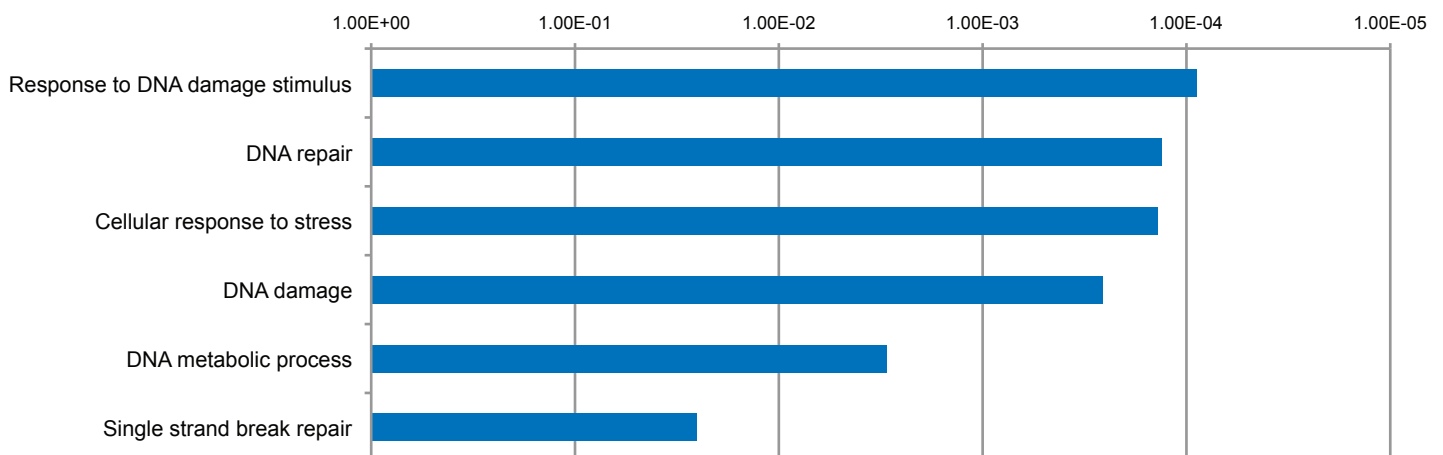

Supplement: Additional file 4 — DAVID Gene ontology analysis on proteins enriched for C, 5mC and 5hmC on the Pax6 probe. 5fC binding proteins showed no significant term enrichment. Results are expressed with their corresponding Benjamini-corrected P value. [file gb-2013-14-10-r119-S4.pdf]

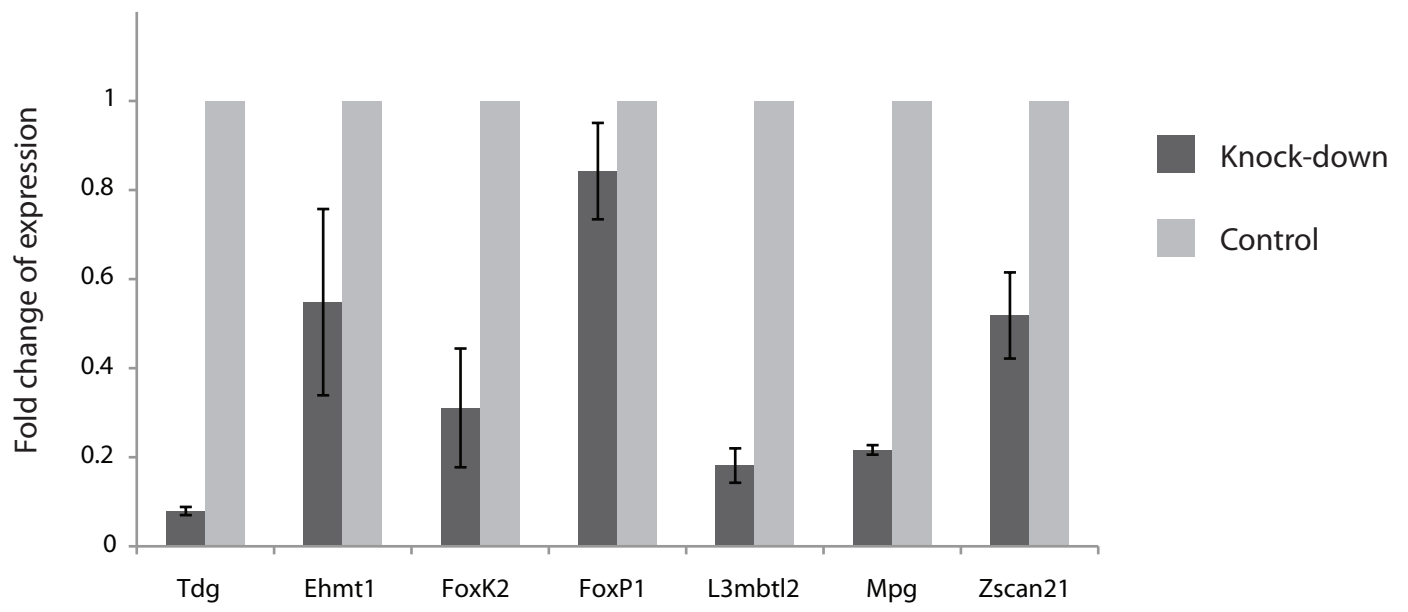

Supplement: Additional file 5 — Knockdown efficiency. Bar plots showing knockdown efficiency in mESC. Dark grey bars indicate mRNA levels in the knockdown samples, light grey in the control samples (transfected with non-targeting siRNA). [file gb-2013-14-10-r119-S5.pdf]
